# A flexible Bayesian method for detecting allelic imbalance in RNA-seq data

**DOI:** 10.1186/1471-2164-15-920

**Published:** 2014-10-23

**Authors:** Luis G León-Novelo, Lauren M McIntyre, Justin M Fear, Rita M Graze

**Affiliations:** Department of Mathematics, University of Louisiana at Lafayette, 70503 Lafayette, LA USA; Department of Molecular Genetics and Microbiology, University of Florida, 32611 Gainesville, FL USA; Department of Biological Sciences, Auburn University, 101 Rouse Life Science Building, 36849 Auburn, AL USA

**Keywords:** Allelic imbalance, Allele-specific expression, RNA-seq, Systematic error, Bayesian model

## Abstract

**Background:**

One method of identifying *cis* regulatory differences is to analyze allele-specific expression (ASE) and identify cases of allelic imbalance (AI). RNA-seq is the most common way to measure ASE and a binomial test is often applied to determine statistical significance of AI. This implicitly assumes that there is no bias in estimation of AI. However, bias has been found to result from multiple factors including: genome ambiguity, reference quality, the mapping algorithm, and biases in the sequencing process. Two alternative approaches have been developed to handle bias: adjusting for bias using a statistical model and filtering regions of the genome suspected of harboring bias. Existing statistical models which account for bias rely on information from DNA controls, which can be cost prohibitive for large intraspecific studies. In contrast, data filtering is inexpensive and straightforward, but necessarily involves sacrificing a portion of the data.

**Results:**

Here we propose a flexible Bayesian model for analysis of AI, which accounts for bias and can be implemented without DNA controls. In lieu of DNA controls, this Poisson-Gamma (PG) model uses an estimate of bias from simulations. The proposed model always has a lower type I error rate compared to the binomial test. Consistent with prior studies, bias dramatically affects the type I error rate. All of the tested models are sensitive to misspecification of bias. The closer the estimate of bias is to the true underlying bias, the lower the type I error rate. Correct estimates of bias result in a level alpha test.

**Conclusions:**

To improve the assessment of AI, some forms of systematic error (e.g., map bias) can be identified using simulation. The resulting estimates of bias can be used to correct for bias in the PG model, without data filtering. Other sources of bias (e.g., unidentified variant calls) can be easily captured by DNA controls, but are missed by common filtering approaches. Consequently, as variant identification improves, the need for DNA controls will be reduced. Filtering does not significantly improve performance and is not recommended, as information is sacrificed without a measurable gain. The PG model developed here performs well when bias is known, or slightly misspecified. The model is flexible and can accommodate differences in experimental design and bias estimation.

**Electronic supplementary material:**

The online version of this article (doi:10.1186/1471-2164-15-920) contains supplementary material, which is available to authorized users.

## Background

Sequence polymorphisms which impact gene expression have been identified as an important factor in human disease (Reviewed in [1–6]); explaining phenotypic differences between individuals (e.g., drug response [7]; biometric traits [8]) and species (e.g., ecological and reproductive traits [9–18]). A variety of experimental designs and analytical methods have been employed to identify the genetic basis of regulatory variation, finding abundant variation in both *cis* and in *trans* regulatory mechanisms [19–32].

In this study we focus on analytical approaches for the analysis of allelic imbalance (AI), a common method used to identify genetic differences in gene regulation. Allelic imbalance occurs when regulatory processes result in different steady-state transcript levels for the two alleles (within a single individual). Genetic differences in the regulation of transcript abundance for a focal gene can arise from regulatory sequence variation occurring within regulatory regions of that gene (*cis* effects) or in regulatory or coding regions of *trans* acting factors (*trans* effects) or through indirect or epistatic effects. The two alleles in a diploid individual are expressed in a common cellular environment. Alleles expressed in a common cellular environment can differ in regulatory sequence, but share a common pool of *trans* acting factors. Therefore, allelic imbalance between alleles in a common cellular environment reveals functional differences between alleles in *cis* regulatory regions [20, 22]. While comparing the same allele in different cellular environments (e.g., between genotypes) reveals differences in *trans* regulation because *cis* regulatory elements are identical while *trans* environments differ.

Early studies of AI focused on a limited number of genes and a few genotypes (e.g., parental genotypes and their F1 progeny, [22, 29]). Different technologies have also been employed, including custom platforms [33], SNP detection [20, 22] and arrays [29, 34]. Currently, the technology most frequently used to asses allele-specific expression is RNA-seq (e.g., [32, 35–38]).

The null expectation, when there is no AI, is that the two alleles are expressed equally (termed allelic balance or AB throughout). That is, the proportion of the total expression level contributed by the maternal/paternal allele is equal to a half. A common approach to analysis of allelic imbalance in RNA-seq data is use of a binomial or chi-square test to determine if allele-specific read counts depart from this expected proportion [32, 35, 39, 40]. However, these tests do not necessarily have the correct error variance [41–45]. Bayesian models have been proposed to improve estimates of allele-specific expression and/or to identify allelic imbalance [46–48].

**Figure 1:**
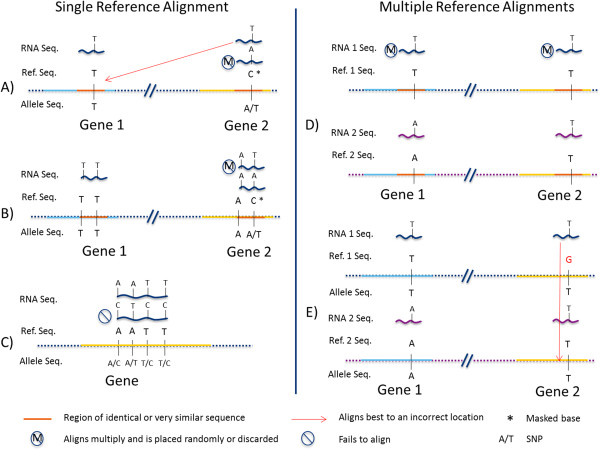
**Sources of error in read alignments and allele-specific read counts contribute to bias in estimation of ASE and AI.** Here we consider error originating from sequence similarity in the genome (e.g., repeats and duplications) and hidden variation (missed or false SNPs). The examples shown illustrate cases for alignments to a single reference **(A-C)** and to multiple references **(D-E)**. Alignments to augmented references are expected to behave similarly to alignments to multiple references. **A)** Masking SNPs located in regions with strong sequence similarity to other locations in the genome (genome sequence ambiguity) can result in alignment error, the best match in the masked reference may be located in a location other than the true source of the read. **B)** Algorithms that account for multiple mapping can result in allele bias when reads from one of the alleles are discarded or are mapped randomly, while reads from the other allele map to their true source location. **C)** For a single unmasked reference, reads from one of the alleles may not align at all, resulting in bias toward the other allele. **D)** When two references are used (one for each parental genome), differences between the references in genome sequence ambiguity can result in allele bias for the same reason as outlined in B. **E)** Sequencing errors in one reference can result in allele bias when reads from both (identical) alleles align best to the other reference.

These Bayesian methods have primarily focused on proper handling of error variance in the statistical model. However, bias in estimation of AI is an important issue for both intraspecific [35, 49] and interspecific [38, 48, 50] studies. Biases are present when aligning to a single reference, a single reference with SNPs masked, and multiple references; which can result in false positives for AI [35, 49–51]. Bias in estimation of allele-specific expression or allelic imbalance has multiple sources, including sequence differences between reads and reference (missed SNPs/false SNPs), properties of alignment algorithms, genome features that result in ambiguity of read alignments and other technical sources of error (Figure 1) [35, 52, 53].

There are several approaches to dealing with bias in studies of AI, which are not necessarily mutually exclusive. Some analytical methods reduce bias resulting from differences between allele-specific reads and a single reference by the use of multiple strain specific references (e.g., [46, 48]) or by allele augmented references [47, 51]. To account for bias due to properties of the alignment, simulated reads have also been used to filter SNPs or other units of analysis that show bias in alignments [35, 46, 51, 54]. For simple F1 experimental designs, the use of DNA controls works quite well [21, 22, 29] to either filter biased regions [50] or to estimate bias and directly account for bias, technical error variance and biological error variance in the statistical model [48].

DNA sequencing of F1 heterozygotes (DNA controls) is used to determine allele-specific read counts in the case where equal amounts of each allele are present in the sample. If there is bias in the DNA read counts the paternal/maternal proportion of reads will deviate from 0.5. Because the measurements from DNA controls reflect the complete process of allele-specific read assessment, the error can originate from properties of the genome (ambiguity, missed variation), mapping, or sequencing related technical bias. This is in contrast to existing filtering approaches, which only capture sources of bias related to ambiguity and mapping. However, use of DNA controls can be cost prohibitive in intraspecific experiments, where the number of genotypes evaluated is expected to be quite high.

In this manuscript we introduce a Bayesian Poisson-Gamma (PG) model for analysis of allelic imbalance. The PG model is a Bayesian version of Poisson regression. This model can be used when DNA controls are not available through use of a parameter representing bias which is incorporated into the structure of the model. The parameter can be fixed (*q*) or random (*ϕ*) and can be used in conjunction with simulation to account for genome ambiguity and map bias.

## Results and discussion

To compare model performance under different scenarios we generated allele-specific read counts for both RNA and DNA controls from a Poisson distribution (see Additional file 1). While total allele-specific reads are distributed similar to real data, bias and the ratio of the two allele mean counts are specified by the parameters *B* and *R* respectively. We investigated model performance for a previously developed Bayesian negative binomial (NB) model [48], the newly developed Bayesian Poisson-Gamma (PG) model, and a binomial model under three different scenarios: a null expectation data set with no bias and no AI, *B*=0.5 and *R*=1; a null expectation data set with bias and no AI, *B*≠0.5 and *R*=1; and a model with bias and AI, *B*≠0.5 and *R*≠1.

To assess the performance of the PG model relative to the NB model when the bias parameter is random, we incorporate simulated DNA control counts (*ϕ* = *DNA*) into the PG model and consider *ϕ*, assuming the same model that we assume for *p* in the NB model (as in (1) below). To determine the impact of using a fixed versus a random bias parameter, we examined both the PG model with *q* and the PG model with *ϕ*. For the NB and PG models with a random bias parameter, the value is taken from replicate simulated DNA control allele-specific read counts. For comparison, we examine the performance of the PG model with *q* = 1/2. In practice, any single value estimate of bias can be used in the PG model with *q*, under a null expectation of no bias *q* = 1/2 is appropriate.

The type I error rate was examined for the null case where allele-specific read counts are generated with no bias (*B* = 0.5) and no AI (*R* = 1). Type I error is less than 5% in all cases, with the NB and PG models showing similar levels of type I error that are lower than that of the binomial, but all tests are valid in this case (Table 1).

**Table 1:** **Estimate of the type I error rate**

Model	Type I error rate
Binomial	4.9%
NB: *p*=*D* *N* *A*	3.5%
PG: *ϕ*=*D* *N* *A*	3.8%
PG: *q*=1/2	3.2%

Allele-specific counts from DNA and RNA-seq data were simulated with no bias and no allelic imbalance for three replicates each of 10,000 exonic regions and analyzed using the binomial exact test, the random bias parameter NB and PG models that use DNA controls and the PG model with fixed bias parameter, q = 1/2. Even when there is no bias, the Bayesian models have better performance than a binomial exact test.

**Figure 2:**
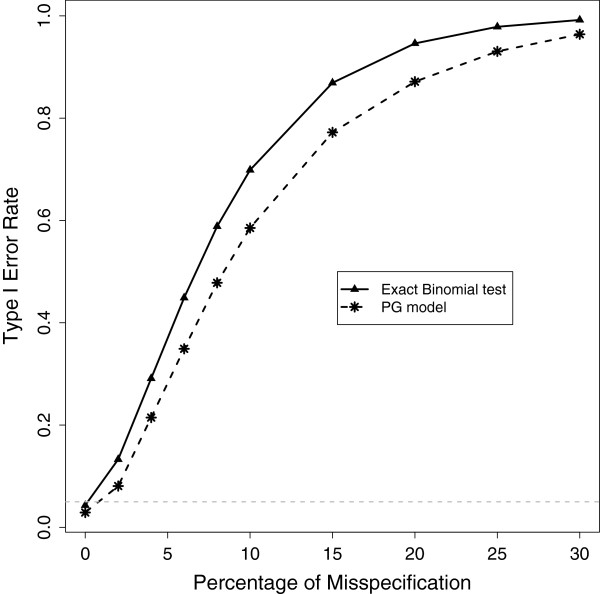
**Type I error rate of the PG model with** ***q*** **= 1/2 and the binomial test, with increasing levels of bias.** The *x* axis is the percentage of misspecification as bias increases above 0.5. That is, *B* = 0.5(1 + *x* *%*) with *x* represented by the the horizontal axis. The horizontal line (grey) through 0.05 is shown for reference.

Both the PG model with a fixed effect of *q* = 1/2 and the binomial exact, assume that there is no bias. That is, the null expectation is equal amounts of reads from the paternal and maternal alleles. However, error variance is handled differently by the two approaches. Is the PG model with *q* = 1/2 different from the binomial test? We compare these models using simulated data sets of allele-specific read counts under a null scenario in which there is bias (*B*≠0.5) and no allelic imbalance (*R* = 1) and simulated data sets with both bias and allelic imbalance (*R*≠1). Comparing the type I error rates for data generated with increasing levels of bias shows that while in both cases the model assumptions are violated and type I error increases with increasing bias, **the PG model always has a lower type I error rate** (Figure 2).

**Figure 3:**
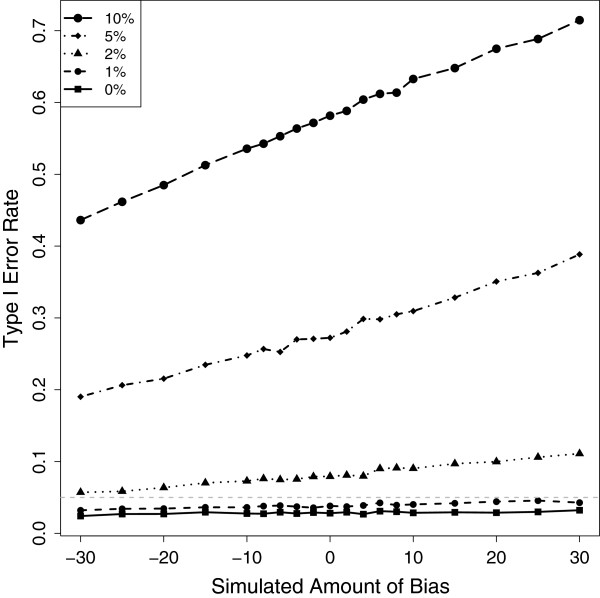
**Simulated amount of bias vs Type I error rate of the PG model with different levels of misspecification.** The *x* axis represents the simulated amount of bias *x* = 2(100)(*B*-0.5) with *B* in the interval (0.35,0.65). The line labeled “0” represents the type I error rate TIER when *q* = (1 + 0*%*)*B*, the line labeled “1%” represents the TIER when *q* = (1 + 1*%*)*B*, and similarly for the lines labeled “2%”, “5%” and “10%”. Note that the smaller the simulated amount of bias is the lower B is and, therefore, the difference between B and the specified *q*, is smaller; hence the lower the TIER. This explains why the TIER increases with the simulated amount of bias. The horizontal line (grey) through 0.05 is shown for reference.

To understand how bias affects model performance, we further investigated the behavior of the PG model with *q* = 1/2 and PG model with *q* = *B*, for *B* = 0.5±10*%error*. The model performs well when there is bias, while the binomial and *q* = 1/2 perform poorly when there is bias (Figure 2; Additional file 1: Figure S1). The model with *q* = *B* controls the type I error rate (2.6%) even when there is bias in the allele-specific read counts. When bias is accounted for but misspecified, the type I error rate depends on the amount of misspecification (Figure 2; Figure 3). Interestingly, when the amount of bias is large misspecification of small amounts (5%) can result in large type I errors. As expected, this appears as slightly asymmetric with respect to the binomial. This is simply due to 1% of 0.65 being a larger absolute amount of bias than 1% of 0.35 (Figure 3). When bias is large and unaccounted for, the PG model with *q* = 1/2 and the binomial can have dramatic type I error rates (Figure 2).

### Using DNA controls or simulated reads to measure bias

What causes bias in estimates of AI? Genome sequence ambiguity and mapping ambiguity can lead to bias, often collectively referred to as map bias. Graze et al. 2012 [48] and Satya et al. 2012 [51] found that the use of separate reference sequences for each allele or augmented single references that contain both alleles reduces map bias. Satya et al. 2012 [51] also found that ambiguity in the reference genome is associated with bias and showed that simple masking of biased SNPs is not sufficient to reduce systematic error in studies of allelic imbalance. Stevenson et al. 2013 [50] observed that changing mapping parameters and filtering can reduce the impact of map bias on estimates of AI when mapping to a single reference.

Simulation studies in which equal numbers of simulated reads from each allele are created and counted, after mapping to each reference, capture genome sequence ambiguity and bias in the mapping algorithm. To incorporate this measure into the PG model, we simulated reads from both a maternal (*D. simulans*) and paternal (*D. melanogaster*) reference. This creates a simulated set of reads that are analogous to sequencing the F1 heterozygote. Mapping these back to both references we counted allele-specific reads corresponding to each allele, using the proportion of allele-specific reads corresponding to the paternal reference as a measure of map bias. Bias was detected approximately 32% of the time in the interspecific read simulation study.

Intraspecific studies are expected to have smaller differences, but are still expected to have regions of genome ambiguity and map bias. Using the DGRP as inspiration [55]. We simulated 94 different F1 genotypes formed by crossing each line to a common tester. Approximately 18% of the time, gene regions were always biased, implicating shared regions of ambiguity among genotypes. In 3% of cases bias was specific to the genotype constructed, implicating a combination of ambiguity and SNP variation among lines. This supports the conclusions of previous studies that bias is likely present in intraspecific studies of AI.

To compare statistical modeling of bias and filtering strategies we examined the behavior of the models using real RNA-seq allele-specific read counts from an interspecific F1 genotype (see Methods for details). We compare a modeling approach with bias measured as the frequency of the paternal allele using allele-specific read counts from DNA sequencing of the same F1 interspecific genotype (PG model with *q* = *DNAcontrols*) with a model that uses an interspecific simulation study to measure map bias (PG model with *q* = *simulation*). Additionally, allele assignment error and genome ambiguity based filtering strategies are investigated.

**Figure 4:**
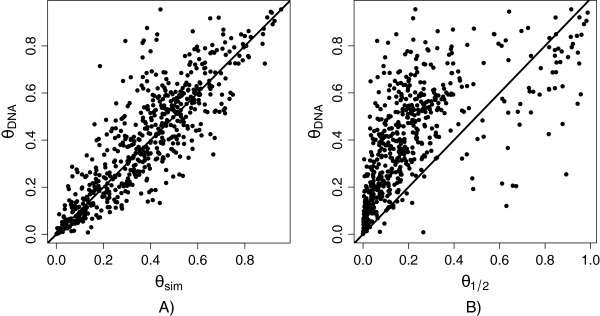
**Comparison of the estimated** ***θ*** **.** The proportion of reads coming from the paternal allele when *q* = *D* *N* *A* *c* *o* *n* *t* *r* *o* *l* *s*, as compared to *q* = *s* *i* *m* *u* *l* *a* *t* *i* *o* *n* **(A)** and *q* = 1/2**(B)**. The *n* = 617 exons with simulated *q* ≠ 0,1 and |*q* - 1/2| > 0.2 are shown.

Read simulation and alignment generally produce smaller estimates of bias than the DNA controls. Often the simulation results in estimates of a half even when the DNA indicates bias. However, using *q* from the simulation study (*q*=*simulation*) does identify a portion of the bias and only rarely does this measure estimate a larger amount of bias then the DNA. This indicates that the PG model with *q* = *simulation* should perform better than the PG model with *q* = 1/2 (Figure 4). Using the DNA controls as “truth”, the proportion of false positives is notably smaller and the number of false positives and false negatives are more balanced in the PG model with *q* = *simulation*, relative to the PG model with *q* = 1/2 (Table 2). The specificity using *q* = *simulation* is larger (0.74) than when using *q* = 1/2 (0.41), but the sensitivity using *q* = *simulation* is smaller (0.69) than when *q* = 1/2 (0.81). Among biased exonic regions there is an exorbitant false positive rate. The false positive rate (FP) is equal to 0.59 (*q* = 1/2), similar to what we observed in analysis of simulated RNA-seq data sets. This is substantially better than binomial (FP = 0.69), but still indicates considerable unaccounted for bias. In comparison, the false positive rate is 0.26 when *q* = *simulation* indicating that using simulated reads to estimate map bias and incorporating this measure into the statistical model dramatically reduces the false positive rate.

**Table 2:** **Model comparisons**

	PG***q*** = ***D*** ***N*** ***A*** ***c*** ***o*** ***n*** ***t*** ***r*** ***o*** ***l*** ***s***		
		AB	AI
PG	AB	0.31	0.18
*q* = *s* *i* *m* *u* *l* *a* *t* *i* *o* *n*	AI	0.11	0.40
		**(a)**	
PG	AB	0.17	0.11
*q* = 1/2	AI	0.25	0.47
		**(b)**	
Binomial	AB	0.13	0.10
	AI	0.29	0.48
		**(c)**	

Comparison of the PG model with q = DNA controls (columns) to (a) the PG model with q = simulation, (b) the PG model with q = 1/2, and (c) the binomial test. The n = 2,230 exons with simulated *q*≠0,1 and |*q*-1/2 |>0.05 are considered.

For those exons where the interspecific F1 simulated read counts do not capture the bias indicated by the DNA controls, we examined other possible sources of the bias. Using a new mapping tool BWA-MEM [56] and the variant caller FreeBayes [57], we identified variants not identified in the initial study [48]. Of the exons where |*q*-1/2|≤0.05 for *q* = *simulation*, there are *n* = 3,923 where *q* = *DNAcontrols* indicates there is no AI but the model based on *q* = *simulation* finds AI. Approximately 38*%* of these have evidence for previously undetected polymorphism while only 27*%* of the *n* = 14,338 where both models indicate no AI is present show evidence for previously undetected polymorphism. This shows that unidentified variants are a source of bias captured in DNA controls, but not in read simulations.

A related source of bias that has been identified as contributing to systematic error in estimation of AI is allele assignment error. False positives and false negatives in SNP calls can result in reads either not mapping at all or to the wrong location, regardless of whether a single or multiple reference approach is used. To identify allele assignment error, RNA derived reads from each parent were aligned onto both the matching and non-matching reference. An exon was considered to show allele assignment error when reads originating from one parent were assigned (based on higher quality alignments to the wrong reference) to the other parent more than 5% of the time. There were 26,896 exons for which both bias as measured by DNA controls and allele assignment could be assessed. There is a positive association between allele assignment error and bias as measured by read simulations and these regions account for a portion of the bias (22%) in DNA controls. However, a large number of exons (2,129 and 5,376 respectively) show allele assignment error, but do not show bias in DNA controls or in simulated interspecific F1 read alignments.

Regions for which parental reads show error in allele assignment may be filtered from the results, as this is expected to contribute to bias. However filtering exons which show allele assignment error reduces the amount of data considered in the analysis, but does not appreciably reduce the false positive rate. Similarly, Satya et al. 2012 [51] noted that regions where alignment simulations found strong bias generally showed genome ambiguity. We assessed genome ambiguity by sequence identity and read mapping. Comparing genome ambiguity with simulated bias we found that in nearly all cases where bias is detected, genome ambiguity is also detected. However, the reverse is not true. There are many regions of the genome which show sequence similarity that do not show strong bias. For approaches which due not account for bias in the statistical model, a filtering strategy based on both ambiguity and allele assignment error has a small effect on the resulting percentage of false positives. However, this eliminates almost a quarter of all the data available. While simulations will not necessarily uncover these biases, filtering based upon them does not improve the overall inferences. Thus, filtering does not seem to be an effective strategy to control type I error rate.

## Conclusions

Even for cases where no control is available, the PG model with *q* = 1/2 is preferable to a binomial test. The PG model had a consistently lower false positive rate than the binomial test. Considering extreme values of AI (greater than 95*%* of reads from the maternal/paternal allele) the PG model with *q* = *DNAcontrol* is the least likely to reject, followed by the PG model with *q* = 1/2 or *q* = *simulation*. The binomial model always rejects in these cases. The PG model with *q* = 1/2 is more conservative even though bias is not corrected or filtered. This is expected if there is extra variance that is not accounted for when using the binomial. This extra variance has been discussed [43, 58] and may well be due to reads being random draws from a distribution rather than the fixed number of trials the binomial assumes.

Accounting for bias by using simulated alignments is a better alternative than using a model in which no bias is assumed. The PG model with *q* estimated from simulated read alignments performs better than using *q* = 1/2, reducing the false positive rate by more than 50%. Simulation captures genome ambiguity and map bias, as well as allele assignment error. When filtering strategies are coincident with bias identified in simulated alignments, they can lower the false positive rate by removing those regions likely to be affected. However, these strategies also remove regions from consideration that do not show allele bias in either simulations or in DNA. Filtering does not provide an advantage over incorporating bias directly into the model and instead removes regions form consideration that can be evaluated using an appropriate model.

Unfortunately, there are additional sources of bias not captured by simulations or filtering strategies. The result is large type I errors. A large source of this bias is likely to be variants that were not initially detected. While this is likely to decrease as variant callers improve, it is worth cautioning that even small amounts of unaccounted for bias result in steep increases in type I error rates.

The flexible Bayesian model proposed here allows for use of DNA controls when they are present. It also has the ability to use a fixed or random parameter for the estimate of bias. If desired, the confidence intervals around *θ* could be widened by allowing for variation in *q* = *simulation* using an external estimate of variability. While we have explored the use of an estimate of bias from read simulations, the model is flexible with respect to other approaches. For example, in the absence of DNA controls or simulations an empirical Bayes approach with a sliding value of bias could be used and the robustness of AI estimates explored across a range of likely values of bias. Alternatively, in cases where there is known bias toward one reference or the other, a single best guess value of the bias could be used, similar in spirit to the skewed binomial test [59]. The model is general enough to accommodate many subtle differences depending on the particular experimental design and approach to estimating bias.

As sequencing costs continue to plummet, population studies of *cis* regulation are on the horizon. Large population studies mean rethinking approaches to evaluating AI, as DNA controls are no longer a viable prospect. Simulations can be effective, but hidden variation can cause significant bias in estimation of AI. Along with improving modeling capabilities, it will be necessary to improve variant callers and to spend more time and effort on large scale population genomic assemblies.

## Methods

### Simulated reads and alignments

Intraspecific read simulations: We simulated 95 *D. melanogaster* genotypes by randomly incorporating 160,000 SNPs into the exonic regions in a single reference sequence (FlyBase 5.51). All possible one hundred base pair reads were simulated from each genotype using a sliding window approach. To create an intraspecific cross using a reference design, one genotype was selected as a reference (Tester), simulated reads from the Tester strain were mixed with each of the remaining 94 genotypes (Lines). The mixed sets of reads were independently aligned to the exon regions in the Tester or Line references using bowtie [-k1 -m1] [60] and LAST [-l 20] [61]. Alignments were compared to determine which reads aligned better to the Tester or Line references and which reads aligned equally well to both the Tester and Line references. Bias towards the line was calculated by taking the number of Line specific reads divided by the total number of allele-specific reads (Tester + Line).

Interspecific read simulations: Starting from the set of strain specific reference exonic sequences [48], all possible 36 bp reads were created from each exon region for each parental strain of the F1 interspecific cross. The number of reads created for each exon region is (L - 36) + 1, where L is the length of the exon region. Exon regions shorter than 36 bp (in either reference) were excluded. Reads were aligned to all exonic sequences in the reference genomes using bowtie [-k1 -m1] and LAST [-l 20]. Reads were separated into three categories based on the highest quality alignment. Reads mapping ambiguously to both references were excluded. If a read mapped with equal quality (and uniquely) to both the maternal (*D. melanogaster*) and paternal (*D. simulans*) references they were assigned to the ‘both’ category. Reads were assigned to the ‘maternal’ category if they aligned best (and uniquely) to the maternal reference. Reads corresponding to the paternal allele were similarly assigned to the ‘paternal’ category. The value used for *q* = *simulation* in the PG model is the proportion of allele-specific reads corresponding to the paternal allele.

### RNA-seq data set

To measure allelic imbalance, reads from two alleles were quantified to estimate allele-specific expression from RNA-seq data for 3 independent replicate samples of RNA from an interspecific F1 hybrid ([48], GEO accession number GSE34591). Briefly, reads were aligned to species references (denoted as maternal and paternal) that were specific to the genotypes used in the experiment. Each reference contains the exonic sequences for one of the parents of the F1 genotype. For each exon, reads contributed to the allele-specific count for each allele (maternal or paternal) when they aligned better to the corresponding reference.

### DNA-seq data set

To control for bias introduced by alignment error or by other technical sources, genomic DNA from the same F1 genotype was collected ([48], SRA accession number SRA048616). Allele-specific read counts for each exon were quantified for DNA as for the RNA data. Inferences from the DNA controls and RNA-seq data are used as the basis by which to compare other models and approaches.

### Identifying ambiguity in the genome

Genome ambiguity was assessed using both sequence identity and read mapping. For FlyBase 5.26, there were 726 exonic regions with an identical sequence to at least one other exonic region. These 726 regions could be grouped into 224 sets of identical sequences. A unique reference was created with all unique exonic regions and only a single representative from each of the 224 identical sets. Next we identified regions where alignment algorithms would have difficulty uniquely placing reads. All possible 36-mers were simulated from the unique reference using a sliding window approach. Some exonic regions (1,261) could not be simulated because they were less than 36 bp. Simulated reads were aligned back to the reference uniquely using bowtie [-k1 -m1]. Ambiguous reads were then re-aligned using bowtie [-k1 -a] placing an ambiguous read at all locations that it mapped. In comparisons of filtering strategies, an exonic region was considered ambiguous (6,229) if there was at least 1 ambiguous read aligning. Ambiguous regions were also identified using BLAST as the alignment algorithm or with the genome mappability analyzer (GMA) [62]. Bowtie, BLAST, and GMA all gave similar results. This process was applied to both species specific references.

### Identifying allele assignment error

Parental RNA from both maternal (*D. melanogaster*, n = 6) and paternal (*D. simulans*, n = 3) lines ([63], GEO accession number GSE54069) were aligned to updated genotype-specific references [48]. Reads were aligned using a multiple step alignment process. First reads were aligned uniquely using bowtie [-k1 -m1]. Unaligned and ambiguous reads were then quality trimmed removing low quality ends. Trimmed reads were then aligned uniquely a second time using bowtie [-k1 -m1]. Finally unaligned and ambiguous reads were aligned uniquely using LAST [-l 20]. For each sample, reads were assigned to a genotype based upon the highest quality alignment. Reads mapping equally well to both genotype-specific references were assigned to a “both” category. In comparisons of filtering strategies, an exonic region was considered to show evidence of allele assignment error when greater than 5*%* of the reads aligned better to the wrong reference.

### Model 1- binomial test

Let *θ* be the unknown proportion of reads from the paternal allele and let *n* be the total number of reads aligning to the exon. This is the standard binomial test of the null hypothesis of no allelic balance *H*_0_:*θ* = 1/2 vs the alternative of allelic imbalance *H*_1_:*θ*≠1/2. Here we reject the null hypothesis if |*z*|>1.965 where  and  is the observed proportion of paternal reads. The advantages of this test are that it is easy to implement and that there are statistical techniques that control for the fact that we are testing multiple tests at the same time. For example, the false discovery rate criterion of Benjamini and Hochberg [64]. Nevertheless, the binomial test does not control for systematic error and bio/tec variation. The standard practice is to compute a measure of bias based on simulated reads and alignments, similarly to *q* = *simulation*, and remove biased regions from the analysis.

### Model 2- negative binomial- with DNA controls, *p*= bias in DNA

Systematic error in inference of allelic imbalance can arise from asymmetry in genetic differences between reads and references used in alignments or differences between references in ambiguity (e.g., CNVs), in combination with the specific alignment algorithm used [35, 51]. Technical sources of systematic error arising from library construction and sequencing may also contribute [52]. Graze et al. 2012 [48] integrated information from a DNA control into the prior for the model used to estimate allelic imbalance in RNA in a Bayesian approach to inference of allelic imbalance. This approach adjusts the estimates of allelic imbalance based on the null expectation for the relative abundance of the two alleles estimated from the DNA controls, *p*. The model that estimates bias using DNA controls estimates the *p* hyper-parameter used in the model that estimates allelic imbalance in RNA-seq data. In the Negative Binomial model the number of reads is random, rather than fixed. For the RNA model: *θ* is the parameter for the proportion of reads coming from the paternal (*D. simulans*) allele, *y*_*i*_ and *x*_*i*_ is the number of RNA reads assigned to the paternal and maternal (*D. melanogaster*) references, respectively, for the replicate *i*. Similarly, for the DNA model:  is the number of paternal assigned reads and  is the number of maternal assigned reads. I (*i* = 1,2,…,*I*) and *I*^⋆^ (*i*^⋆^ = 1,2,…,*I*^⋆^) are the number of replicate RNA and DNA sample respectively. The RNA model is,


and the DNA model is,
1

Here the parameterization of the Negative Binomial distribution is such that, if *η*∼*NegativeBinomial*(*k*,*ε*), then *η*∈{0,1,… } denotes the number of failures before the first *k* successes with probability of success equal to *ε*.

### Model 3- poisson gamma

The PG model can be used when DNA control is not available by using *q* (fixed). Unlike the NB model which incorporates *p* as a prior, the PG model incorporates the parameter *q* (fixed) into the structure of the model. The model can also be specified with *ϕ* (random) if replicate measures of bias are available as for DNA controls. Let *x*_*i*_ and *y*_*i*_ be the maternal and paternal, respectively, RNA reads in the biological replicate *i*, *i* = 1,…,*I*. We assume,
2

Here *μ* is the overall mean, a nuisance parameter. The parameter *β*_*i*_, *i* = 1,…,*I* models the biological replicate variation. *q* is a constant that incorporates the information about the bias into the model. *q* in the PG model plays a role similar to that of *p* in the NB model. When we perform the simulation we make *q* equal to the proportion of simulated reads aligning to the paternal reference.

When we have DNA information we make *q* equal to the proportion of DNA sequencing reads from an F1 genotype aligned to the paternal reference. If DNA information is present, then a random bias parameter *ϕ* can be sampled from the posterior of the DNA model and used as a true value in the RNA model, and *θ* can then be sampled from the posterior, under the RNA model. For example, we sample *ϕ*^*m*^ = *p* from the posterior of model (1) and obtain a posterior sample of size 1, , under model in (2) for every *m* = 1,…,*M*. We flag the exon as in AI if the CI for *α* does not contain 1 (or, equivalently, the CI for *θ* does not contain 1/2). When comparing the PG model and the NB model we follow this approach, using *p* (random) and *ϕ* (random). For fair comparison, we contrast the PG model with *q* = *simulation* (fixed) and with *q* = *DNAcontrols* (fixed). The parameter of interest is the treatment effect, *α*. If *θ* is the “real proportion of reads from the paternal allele”,


So when there is no AI, *α* = 1 and


the parameters *q*, *θ*, *x* and *y* in the Poisson Gamma model play the role of *p*, *θ*, *x* and *y* in the negative binomial model. We give standard priors to the parameters: *μ*∼*Gamma*(*a*_*μ*_ = 1/2,*b*_*μ*_ = 1/2), *β*_1_,…,*β*_*I*_∼*Gamma*(1/2,1/2) and *α*∼*Gamma*(1/2,1/2). Here *η*∼*Gamma*(*a*,*b*) is parameterized such that *E*(*η*) = *a*/*b*.

Note that the model is parameterized to estimate the abundance of one of the alleles (the paternal), rather than the relationship between two alleles. If the bias is in the opposite direction relative to the the paternal allele, then the Type I error rate will be lower than if the bias is in the direction of the paternal allele. If it is likely that a global bias in one direction exists — perhaps due to a difference in reference quality — and the type I error is a greater concern than power, the model should be parameterized such that the allele estimated is the allele that is not favored by bias.

## Availability of supporting data

The data sets supporting the results of this article are available in the following repositories: The F1 interspecific hybrid RNA sequencing data and corresponding parental RNA sequencing data are available from the Gene Expression Omnibus database (GEO) repository with accession numbers GSE34591 and GSE54069, respectively. The F1 interspecific hybrid DNA sequencing data are available from the NCBI Short Read Archive (SRA), accession number SRA048616.

## Electronic supplementary material

Additional file 1: **Allele-specific read count simulation study.** (PDF 103 KB)
