# Supplementary material for: A flexible Bayesian method for detecting allelic imbalance in RNA-seq data
Source: BMC Genomics. 2014 Oct 23;15(1):920. doi: 10.1186/1471-2164-15-920 (PMC4230747; doi:10.1186/1471-2164-15-920)
Supplement: Supplementary file 1 — Additional file 1: Allele-specific read count simulation study. (PDF 103 KB) [file 12864_2014_6642_MOESM1_ESM.pdf]

Additional File: Allele-specific read count simulation study

Luis G. León-Novelo Lauren M. McIntyre Justin M. Fear Rita M. Graze

1 Poisson Synthetic Data

This supplement describes the way Poisson synthetic data were generated and analyzed.

1.1 Data Generation

Let the subindices m and p denote maternal (*D. melanogaster*) and paternal (*D. simulans*), respectively. Data under three simulation scenarios were generated. Each is a variation of the simulation scheme presented below. In order to describe the scheme for generating synthetic data we introduce the following notation:

$$\begin{aligned} x^s_{i,RNA} &= \text{number of (simulated) reads from maternal in the RNA for biorep } i, \\ y^s_{i,RNA} &= \text{number of (simulated) reads from paternal in the RNA for biorep } i, \\ x^s_{i',DNA} &= \text{number of (simulated) reads from maternal in the DNA for biorep } i', \\ y^s_{i',DNA} &= \text{number of (simulated) reads from paternal in the DNA for biorep } i', \end{aligned}$$

$i = 1, \ldots I$, $i' = 1, \ldots I'$. I the total number of RNA biological replications and I' the number of DNA biological replications. Here $I = I' = 3$ as in the real dataset. The superindex s is a reminder that this is simulated data. The sampling distributions for the RNA counts are,

$$x^s_{i,RNA} \overset{\text{iid}}{\sim} \text{Poisson}(B\mu_m) \quad \text{and} \quad y^s_{i,RNA} \overset{\text{iid}}{\sim} \text{Poisson}((1-B)\mu_p), \quad i = 1, 2, \ldots, I, \tag{1}$$

and for the DNA counts are,

$$x^s_{i',DNA} \overset{\text{iid}}{\sim} \text{Poisson}(B\mu_{DNA}) \quad \text{and} \quad y^s_{i',DNA} \overset{\text{iid}}{\sim} \text{Poisson}((1-B)\mu_{DNA}) \quad i' = 1, 2, \ldots, I'. \tag{2}$$

Here B denote the bias parameter ($B = 1/2$ is no bias). In particular, when $B = 1/2$, the expected maternal DNA counts and the expected paternal DNA counts are the same. Let $R = \mu_m/\mu_p$ be the ratio of the RNA maternal mean counts and RNA paternal mean counts after correcting for bias. So $R = 1$ when there is no AI; and, for example, when $R = 2$ the RNA maternal counts have mean twice the paternal counts mean, if there were no systematic bias ($B = 1/2$), or after correcting for it. In all scenarios we randomly draw 10^4 exons from the real dataset. We use the RNA and DNA real counts to define RNA and DNA

mean counts μ_m, μ_p and μ_{DNA} in the simulated datasets. We use the sampling distributions (1) and (2) to generate data for every exon. The full notation is $\mu_{m,g}$, $\mu_{p,g}$, and $\mu_{DNA,g}$ with $g = 1, 2, \ldots, 10^4$, but, to simplify notation, we omit the exon subindex g. For every exon, we define the parameters of the sampling distributions above, (1) and (2), in the following way:

1. Set $\mu_T \equiv \sum_i (x_{i,RNA} + y_{i,RNA})/I$ equal to the total allele-specific reads (maternal real sample RNA mean counts+paternal real sample RNA mean counts). Here $x_{i,RNA}$ and $y_{i,RNA}$ denote the maternal and paternal RNA counts for the biorep i in the real dataset.

2. To mimic the real data set we will generate $I = 3$ counts (corresponding to 3 bioreps) labeled as originated from maternal alleles and $I = 3$ counts labeled as originated from paternal alleles.

3. The maternal allele counts have mean $B\mu_m$ with $\mu_m = a\mu_T$, and the paternal allele counts have mean $(1 - B)\mu_p$ with $\mu_p = b\mu_T$, with a and b positive constants. To mimic the coverage of the real data set in our synthetic data sets, we require $Ba + (1 - B)b = 1$ so that $B\mu_m + (1 - B)\mu_p = \mu_T$. Moreover, we require $a/b = R$ so that $R = \mu_m/\mu_p$. Solving this system of equations we obtain

$$a = Rb \quad \text{and} \quad b = \frac{1}{(R-1)(1-B)+1} \tag{3}$$

4. We simulate DNA data only in Scenario 1, there we describe how we do so.

1.2 Scenario 1: No AI and No Bias

We simulate a dataset with 10^4 exons and no AI, *i.e.* $R \equiv 1$, and no bias, *i.e.* $B = 1/2$, or, equivalently, with $a = b = 1$ in (3). In addition, we generated DNA simulated counts as we did with the RNA counts but, to mimic the real data DNA coverage, setting $\mu_{T,DNA} \equiv \sum_{i'} (x_{i',DNA} + y_{i',DNA})/I'$ (defined as μ_T in 1 Subsection 1.1 but with the DNA, instead of RNA, data) equal to the maternal real sample DNA mean counts+paternal real sample DNA mean counts, we then set $\mu_{DNA} = (1/2)\mu_{T,DNA}$.

We analyze this data set with

1. Negative binomial model in [1] with the parameter p random (plays the same roll of q or ϕ in the PG model) .

2. PG model with ϕ (random). Remember that, for comparison, we consider ϕ assuming the same DNA model that the negative binomial model in [1].

3. Binomial exact test.

4. PG model with $q = 1/2$ (fixed).

We estimate the type I error rate (TIER) of each procedure as the proportion of exons where the procedure finds significant AI. The binomial exact test concludes an exon shows AI if its p-value is less than 0.05. The PG and Negative binomial models conclude an exon shows AI if the central 95% credible interval for the proportion of paternal reads does not contain 0.5. Table 1 in the paper shows the results.

1.3 Scenario 2: No AI and Bias

We simulate a dataset with 10^4 exons and no AI, *i.e.* $R \equiv 1$. To generate bias count samples, we set $B = 0.45$ for the first 5000 genes and $B = 0.55$ for the last 5000 genes.

We analyze this data set with

1. Binomial exact test.
2. PG model with $q = 1/2$ (fixed).
3. PG model with $q = B$ (fixed).

We estimated the TIER for this dataset. The results are reported in the paper.

Additionally, we generated data sets with $B = 0.35, 0.375, 0.4, 0.425, 0.45, 0.46, 0.47, 0.48, 0.49, 0.50$ for all exons, analyze the data set with the procedures 1-3 above and, also, with the PG model with different levels (1,2,5 and 10%) of misspecification of B. This is, we analyze these data sets with PG models with values of $q = d \times B$ with $d = 1.01, 1.02, 1.05$ and 1.1. This allows us to examine the results to those for the PG model with $q = 1/2$ and the binomial exact test in terms of TIER (See Figures 2 in the main manuscript) and compare the TIERs of the PG model at different levels of misspecification (See Figure 3 in the main manuscript).

1.4 Scenario 3: AI and Bias

We simulate a dataset with 10^4 exons with AI, setting $R = 1.5$ and with systematic bias, setting $B = 0.45$ for the first 5000 exons and $B = 0.45$ for the last 5000 genes. That is, we generate allele-specific counts with poisson $\mu_m = a0.45 * \mu_T$ and $\mu_p = b(1 - 0.45) * \mu_T$ for the first 5000 genes, and $\mu_m = a(0.55) * \mu_T$ and $\mu_p = b(1 - 0.55) * \mu_T$ for the last 5000 genes. The constants a and b are computed according to (3).

We analyze this data set with

1. Binomial exact test.
2. PG with $q = 1/2$ (fixed).
3. PG with $q = B$ (fixed).

We estimate the power of each one of the procedures as the proportion of exons that the procedure flags as in AI. We compare the PG model and the binomial exact test in terms of the receiver operating characteristic (ROC) curve (Additional file 1: Figure S1).

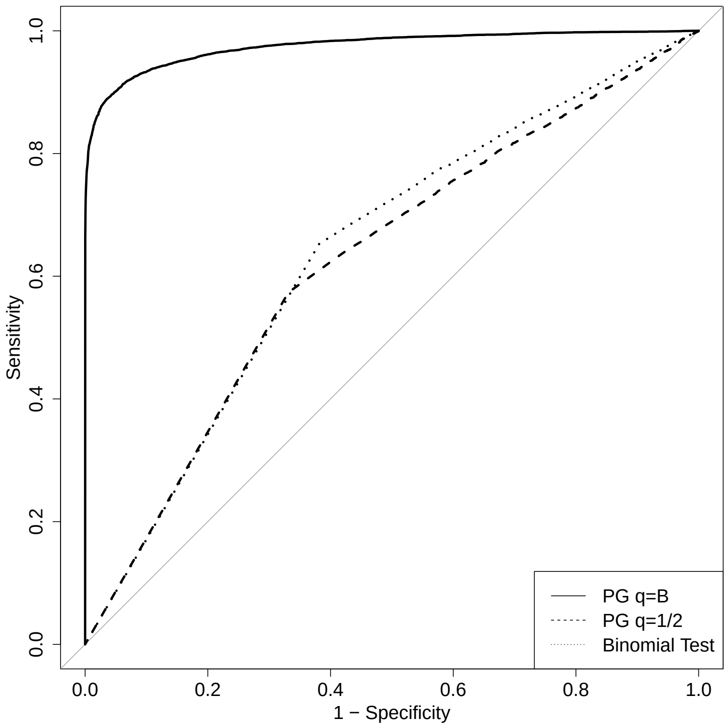

Additional file 1: Figure S1: The datasets of scenarios 2 and 3 were merged to obtain a dataset with 10^4 exons not in AI and 10^4 exons in AI. For each exon we compute the "Bayesian pvalue"$= 2\min\{P[\alpha > 1 \mid data], P[\alpha < 1 \mid data]\}$, this is, the minimum value, such that the central $1-$"Bayesian pvalue" credible interval does not contain $\alpha = 1$ (See [2]), remember α is a parameter in the PG model. We used these "Bayesian p-values" under the PG model with $q = B$ and under the PG model with $q = 1/2$ as classifiers in the ROC curve. We used the p-values of the exact binomial test as classifier for a third ROC curve.

References

[1] Graze R, Novelo L, Amin V, Fear J, Casella G, Nuzhdin S, McIntyre L: **Allelic imbalance in Drosophila hybrid heads: exons, isoforms, and evolution**. *Molecular biology and evolution* 2012, **29**(6):1521–1532.

[2] Thulin M: **Decision-theoretic justifications for Bayesian hypothesis testing using credible sets**. *Journal of Statistical Planning and Inference* 2014, **146**:133–138.
